# Indicators of Quality of Clinical Care for Type 2 Diabetes Patients in Primary Health Care Centers in Qatar: A Retrospective Analysis

**DOI:** 10.1155/2019/3519093

**Published:** 2019-12-05

**Authors:** Saleh Attal, Mohamed H. Mahmoud, Muna Taher Aseel, Ady Candra, Paul Amuna, Mohamed Elnagmi, Mostafa Abdallah, Nahed Ismail, Ahmed Abdelrazek, Dia Albaw, Abdulsalam Albashir, Hisham Elmahdi

**Affiliations:** ^1^Family Medicine Residency Program, Primary Health Care Corporation, West Bay Training Center, Doha, Qatar; ^2^Research Department, Primary Health Care Corporation, Doha, Qatar

## Abstract

**Background:**

Despite the high prevalence of type 2 diabetes mellitus in Gulf countries, standards of diabetes care at the primary care level have not been widely studied.

**Aim:**

To compare the results of diabetes clinical indicators from the American Diabetes Association (ADA) 2017 guidelines to the reference benchmarks in the Behavioral Risk Factor Surveillance System.

**Materials and Methods:**

A cross-sectional analysis of electronic medical records in 643 randomly selected adult patients with type 2 diabetes was undertaken. A checklist enabled the collection of sociodemographic, clinical, biochemical, and quality measurement data. Data were analyzed using Stata 9.0. The chi-squared test was used to compare two or more proportions.

**Results:**

There were 643 patients (male = 60.3%; female = 39.7%), and the majority (71.7%) aged between 40 and 64 years. Common comorbidities were dyslipidemia (72.3%), hypertension (70%), obesity (50.1%), and preobesity (overweight) (37.9%). Over 15% were smokers. The most commonly prescribed diabetes medications were metformin (89.9%), dipeptidyl peptidase-4 inhibitors (61.1%), and sulfonylureas (49.3%). Only 35.5% (*p* < 0.0001) of patients met the reference glycated hemoglobin (HbA1c) cutoff level of 7.0%. The reference level for blood pressure control was met by 70.2% (*p* < 0.0001) and for low-density lipoprotein cholesterol, 73.8% (*p* < 0.0001). Albuminuria was present in 39.2%, and very low vitamin D level (<20 ng/ml) in 39.1%. Most patients had annual foot (89.6%, *p* < 0.0001) and eye (72.3%, *p* < 0.0001) examinations. Only 39.9% had referrals for dietary counseling, and there were lower rates of referrals and uptake for pneumococcal, influenza, and hepatitis B vaccines. Most (76.2%) did not have screening for depression.

**Conclusion:**

The majority of the results met the ADA standards, while glycemic control, dietary counseling, and screening for depression were poor in comparison to the standards. Continuing education for clinicians, patient education for self-management, and targeted weight management are recommended.

## 1. Introduction

Diabetes mellitus currently presents one of the most significant burdens on public health. It is a chronic disease requiring comprehensive medical care combined with different risk-reduction strategies, not limited to glycemic control [1]. In 2015, over 415 million adults (aged 20–79 years) had diabetes, consuming 12% of global health expenditure; their number is predicted to reach 642 million by 2040 [2].

In 2012, diabetes directly contributed to 1.5 million deaths globally, and uncontrolled blood glucose caused another 2.2 million deaths indirectly, through elevated cardiovascular risks and other diseases [3]. In Qatar, the prevalence of diabetes among Qatari adults was estimated at 16.7% in 2012, higher in women, and peaked in the age group 40–49 years (31.2%) [4]. Prevalence is expected to reach 24% and to consume 32% of total health expenditure by 2050 [5].

Some regional studies have reported modest to low compliance with international benchmarks in the level of care provided to patients with diabetes [6, 7]. The Healthcare Effectiveness Data and Information Set (HEDIS) results also showed variable levels of comprehensive diabetes care in the USA [8]. Selecting the correct indicators for diabetes care is essential to optimizing care for patients. According to international experts, three main criteria are crucial in the selection of indicators: the process of care, proximal outcomes, and distal outcomes [9]. A 10-year case-control study utilizing a comprehensive diabetes management program showed significant improvements in the healthcare process and outcomes for the studied patients with diabetes [10].

Our primary goal was to compare the results of diabetes clinical indicators, adopted by the Ministry of Public Health in Qatar from the American Diabetes Association (ADA) 2017 guidelines [11] to the reference benchmarks in the Behavioral Risk Factor Surveillance System (BRFSS), which is an annual nationwide telephone surveillance survey published by the Centers of Disease Control and Prevention (CDC). BRFSS data are useful in health promotion and disease prevention programs and are gathered from all 50 states and US territories, reporting the modifiable risk behaviors and different factors affecting mortality and morbidity in the population [12]. Secondarily, the study also aimed to measure the prevalence of other comorbidities among our diabetes patient cohort and describe the number and types of medications used by patients with diabetes.

## 2. Materials and Methods

### 2.1. Methods and Setting

A retrospective cross-sectional study was conducted of the electronic medical records (EMR) of adult patients with type 2 diabetes attending noncommunicable diseases (NCD) clinics at the Primary Health Care Corporation (PHCC) in Qatar in June 2017 until December 2017. PHCC is the main public provider of primary care for the whole population living in Qatar. PHCC patronizes a total of 27 health centers spread across Qatari cities, and every individual living in Qatar must be assigned to one of those health centers to receive their health care needs. Thus, PHCC covers all members of population in Qatar. At the time of the study, a total of 23 primary health centers (PHCs) were equipped with EMR (namely, the Cerner Millennium® patient administration system). The PHCs provide outpatient services across clinical disciplines, run by clinical teams led by family physicians who are trained in diabetes management including insulin initiation. The NCD clinics offer comprehensive care for chronic illnesses such as diabetes as well as other comorbidities and complications. Patients follow up their conditions with physicians in a period of 2-3 months and are being prescribed their medications based on the assessment with a secondary care referral pathway for complicated type 2 diabetes cases and type 1 diabetes. Most of the diabetes medications were available and publicly funded. During the study period, however, GLP-1 and SGLT-2 were just introduced in a few primary care centers, and thus, patients requiring any of these medications were referred to the secondary care.

### 2.2. Subjects

All patients 18 years or older (diagnosed with type 2 diabetes) were eligible for inclusion if they had at least two NCD clinic visits in 2017. Patients younger than 18 years, those with type 1 diabetes, and women with gestational diabetes were ineligible. The diabetes type 2 patients were identified and labelled by their physicians using the ICD-10 code, and to confirm the labeling, we look for their HbA1c and fasting blood glucose results [1].

### 2.3. Sample Size Calculation and Sampling Techniques

Following an initial review of EMR data from 20,777 patients from 23 PHCs, a total of 13,684 patients' records were eligible for inclusion in the sampling frame. Applying Cochran's formula for sample size calculation for proportions, a minimum sample size of 217 [13] was calculated based on a prevalence of 17% [5], precision of 0.05, and a 95% confidence interval. For greater precision, we increased the sample size to 650.

Sample selection was by a multistage random sampling technique. It was initially determined that each PHC would contribute 4.75% of the sample, stratified according to the size of the patient caseload to ensure proportionate distribution. Individual patient records were selected by systematic random sampling. Seven patients who were labelled as having diabetes type 2 but upon confirmation from HbA1c and fasting blood glucose results were prediabetes and were excluded from the final sample (*n* = 643).

### 2.4. Study Variables

We developed a checklist to collect EMR data, comprising the following: (1) sociodemographic variables including patient's age, sex, nationality, marital status, educational level, and employment; and (2) clinical and biochemical information including medical history, body weight and height, blood pressure (BP), fasting serum lipids, glucose, HbA1c, and vitamin D levels.

Body mass index (BMI) derived from body weight and height was used to assess preobesity/overweight (BMI > 25 and <30) and obesity (BMI ≥ 30). Cutoff points set for indicators of risk included glycemic control, HbA1c <7.0%; lipid control, low-density lipoprotein cholesterol (LDL-C) <2.6 mmol/L; BP control, <140 mmHg (systolic) and <90 mm Hg (diastolic); vitamin D level of 30 ng/ml; presence of albuminuria; and retinopathy [1].

A checklist comprising 15 indicators of comprehensive care based on the ADA 2017 guidelines [1] was used to assess the level of diabetes care. Care process indicators included dietary and exercise counseling and referral for these; annual foot and ophthalmic examinations; records of annual HbA1c, LDL-C, and albuminuria checks; up-to-date records of BP and BMI; screening for depression; influenza (flu), pneumococcal (PCV), and hepatitis B (Hep B) vaccination; and information on diabetes-specific and comorbidity-related medications, e.g., aspirin, statins, antihypertensives, and vitamin D supplementation. The results of PHCC diabetes care clinical indicators were compared to the Behavioral Risk Factor Surveillance System (BRFSS) [11] targets.

### 2.5. Data Analysis

Data were collated in Epi Info™ 7.0 [14] and analyzed in Stata 9.0 [15]. Descriptive statistics (mean, standard deviation (SD)), frequency distribution (percentages/proportions), and bivariate analysis (chi-squared test) were computed and used as appropriate. Student's *t*-test was used to compare the means of two continuous variables. A *p* value ≤0.05 was considered statistically significant.

### 2.6. Quality Control Measures

A data extraction sheet/checklist was developed based on the HEDIS measures and ADA standards and used for assessing the diabetes care process and levels of care [1, 8]. Two authors were assigned data handling for consistency. The principal authors reviewed data entry for accuracy. For reliability, all biochemical measurements were undertaken using the same regularly calibrated standardized equipment in all laboratories.

## 3. Results

There were 643 patients in a male to female ratio of approximately 60 : 40%. The majority (71.7%) of patients were aged 40–64 years. Of the 643 patients, 23.8% were Qataris, 29.9% non-Qatari Arabs, and 46.3% non-Arabs. Patients lacking complete documentation of sociodemographic and lifestyle variables were as follows: marital status 406, financial status 625, educational level 607, employment status 236, and smoking status 136. Over 15% were documented as smokers (Table 1).

**Figure 1:**
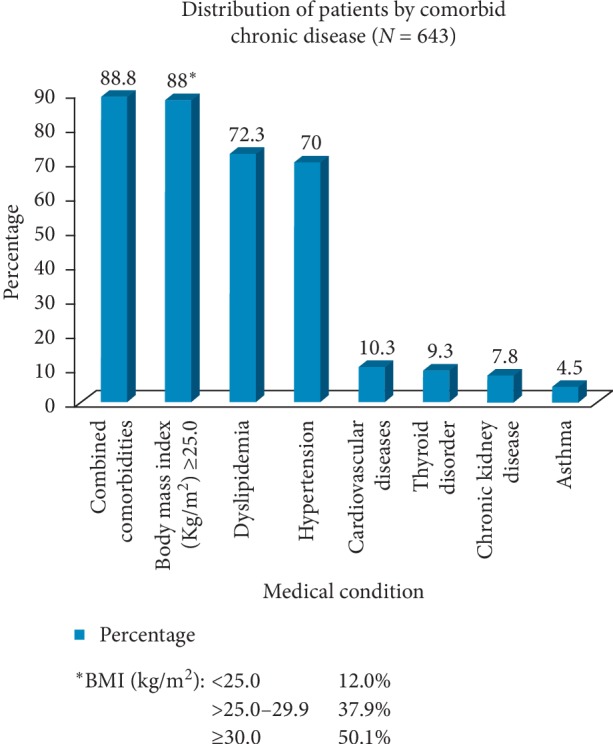
Distribution of patients by comorbid chronic disease (*N* = 643).

Most patients (88.8%) had at least one comorbidity, the most common of which were dyslipidemia (72.3%), hypertension (70%), obesity (50.1%), and preobesity/overweight (37.9%). Another 10.3% and 7.8% had coronary artery and chronic kidney diseases, respectively (Figure 1). While 51.7% were taking three or more diabetes medications, 1.4% were not taking any medication. The most common diabetes treatments were metformin (89.9%), dipeptidyl peptidase-4 (DDP4) inhibitors (61.1%), sulfonylureas (49.3%), and insulin (27.8%), with a small percentage of patients taking thiazolidinedione (10.1%), glucagon-like peptide-1 receptor (GLP-1) agonists (2.3%), sodium-glucose cotransporter-2 (SGLT-2) inhibitors (1.9%), and meglitinides (0.8%). Other pharmacological treatments for comorbidities included statins (79.2%), angiotensin-converting enzyme (ACE) inhibitor/angiotensin receptor blockers (ARBs) (67.7%), aspirin (35.8%), and vitamin D (68.7%) (Table 2).

Only 39.9% of patients were referred for dietary counseling, and dietitians and physicians counseled 37.6% of patients, while 62.2% received exercise counseling from physicians and nurses. Most patients (89.6%, *p* < 0.0001) underwent annual foot examinations, and 72.3% (*p* < 0.0001) received annual dilated eye examinations by an ophthalmologist. However, 12.8% received no referral for routine retinopathy screening. Most patients had had their LDL-C level and urinary albuminuria checks during the previous year (92.8%, *p* < 0.0001; 80.9%, *p* < 0.0001, respectively), while 47.4% (*p* < 0.0001) had at least two HbA1c checks over the same period. Flu, PCV, and hep B vaccinations were recorded for 40.9% (*p* < 0.0001), 43.9% (*p* < 0.0001), and 7.5% of patients, respectively. Vaccinations for flu, PCV, and hep B were ordered but not received in 10.3%, 4.2%, and 4.8% of patients, respectively. BP and BMI were recorded for 97.5% and 96.6% of patients, respectively. A majority (76.2%) of patients lacked records of screening for depression (Table 2).

Despite the level of apparent engagement with service providers, only 35.5% of patients attained the desired level of glycemic control (HbA1c < 7.0%). A further 27.7% had HbA1c between 7.0 and 7.9%. There was poor glycemic control in almost 30% of patients, with 20.9% recording HbA1c ≥ 9.0%. A majority of patients (70.2%) had BP < 140/90 mmHg from their last NCD clinic visit and most (73.8%) had LDL-C levels <2.6 mmol/L. Albuminuria was present in 39.2% of patients, retinopathy in 8.7%, and vitamin D level <20 ng/ml in 39.1% (Table 3).

PHCC results showed the percentage of patients receiving annual eye examinations, comprehensive foot examinations, and screening for albuminuria as 72.3%, 89.6%, and 80.9%, respectively. The combined total of patients who were nonsmokers and had controlled BP, LDL-C, and HbA1c was 12.4% (*p*=0.0026) (Table 3).

## 4. Discussion

In this study, people with diabetes were 60.3% male compared to 39.7% female. This conforms to the sex ratio in the unique population pyramid of Qatar where among non-Qataris, the sex ratio is 79.9% male compared to 21.1% female, while among Qataris, the sex ratio is 49.8% male compared to 50.2% female. The demographic imbalance in the male-female ratio in non-Qataris is due to the high influx of male expatriate workers [16]. The previous study on diabetes in Qatar [4] showed a majority of women as it included only Qataris, matching a similar study conducted in Dubai [6].

While the high proportion (76.2%) of non-Qatari patients reflects the population, the proportion of Qatari adults in the sample (23.8%) is almost double the total share of Qataris in the national population [16], suggesting a higher prevalence of diabetes among Qataris. Adults aged 40–64 years represented the majority (71.7%) in this study. This conforms to a systematic review of the prevalence of type 2 diabetes in the Gulf Arab states which shows an increased prevalence of type 2 diabetes with the advancing age [17].

The prevalence of documented smoking status was 15.2%, and those smokers were more likely to report poor health and perform poorly in diabetes management indicators [18]. Other sociodemographic factors such as marital status, educational level, employment, and financial status were poorly documented; this should be improved in the future (Table 1).

Most (88.8%) of our diabetes patients had at least one comorbidity, consistent with the study by Pantalone et al. that concluded type 2 diabetes patients have a high number of comorbidities [19]. In our study, dyslipidemia was the most prevalent comorbidity at 72.3%. The percentage was based on the number of patients labelled as having dyslipidemia, following the ADA guidelines to prescribe statins as the primary intervention to prevent cardiovascular disease (CVD). Due to the mislabeling of some patients, the percentage taking statins was higher (79.2%), as we were unable to subcategorize patients based on whether they take statins for dyslipidemia or for primary prevention. This needs to be explored more in future studies. Our study showed lower rates of hypertension and CVD (70% and 10.3%, respectively) than Pantalone's study (87.2% and 22.3%, respectively) [19].

Our study shows a high prevalence of overweight subjects at 37.9%, comparable to 37.4% in the recent study [20]. In addition, a high prevalence of obesity at 50.1% of all age groups is comparable to recent Qatar and US studies showing 41.4% [5] and 39.8% [21] prevalence of obesity, respectively.

Taking into account these comorbid conditions that present with diabetes, there has been a renewed emphasis on applying evidence-based comprehensive medical evaluation. This approach departs from the traditional approach of focusing mainly on the treatment of single diseases [1, 22].

In keeping with findings in similar studies [6, 18], metformin was the most prescribed diabetes medication at 89.9%. The second most prescribed medication comprised DDP-4 inhibitors (61.1%), and the third most prescribed medication comprised sulfonylureas (49.3). This contrasts with two studies in which sulfonylureas were the second most prescribed [6, 18]. The DDP-4 inhibitor has a low risk of hypoglycemia and is weight neutral; as the cost is paid by the public health care system in Qatar, it is preferable to sulfonylureas. Pantalone's study [19] showed that prescription of insulin increased from 15.2% (2008) to 18.8% (2013), compared to 27.8% insulin prescription in our study. Physicians and patients play a role in the decision to start insulin, and patients with uncontrolled diabetes, in particular, will require increased insulin usage in the future. Smaller numbers of patients were taking thiazolidinedione (10.1%), GLP-1 agonists (2.3%), SGLT-2 inhibitors (1.9%), and meglitinides (0.8%). The low percentages of both GLP-1 agonists and SGLT-2 inhibitors were expected, as both were newly introduced at the start of the study, but their usage is expected to increase significantly following the updated guidelines. In this study, 15.8% of patients were taking four or more medications.

Other medications for comorbidities include statins (79.2%), ACE inhibitor/ARBS (67.7%), aspirin (35.8%), and vitamin D (68.7%). The high percentage of patients taking statins may be explained by the latest guidelines' emphasis on the importance and cost-effectiveness of statins for adults with diabetes. ACE inhibitors/ARB were prescribed for hypertension and/or prevention of microalbuminuria in patients with diabetes during the study period. Preliminary labeling of some patients as hypertensive on the first encounter, despite requiring further confirmation before starting medication, had led to a higher percentage of patients being classified as having hypertension. Aspirin was prescribed for primary and secondary prevention in 35% of patients; as the guidelines are changing, the figure is expected to change accordingly. Moderate-to-severe vitamin D deficiencies (<20 ng/mL) were recorded in 39.1% of our patients from the overall population, compared to 61% in the general population of Qatar [23]. Of patients in our study, 68.7% were prescribed vitamin D supplements. A study showed that type 2 diabetes can be prevented by high vitamin D status [24]. However, the topic of vitamin D and type 2 diabetes is still contentious and the largest study thus far, the D2d study, was unable to confirm that assumption [25].

Nutritional counseling remains a challenge in clinical practice despite the ADAs endorsement of medical nutrition therapy to provide a flexible, individualized approach to patients' dietary control [1]. We found low rates of referral by physicians (39.9%) and counseling received from certified dietitians and NCD physicians (37.6%). The latter are not trained in nutrition education and so may be inadequately equipped to offer the best evidence-based advice. The same issue is applicable to exercise counseling (62.2%), which despite moderate results was not conducted by professional physical therapists. Diet and exercise are two crucial lifestyle modifications required to achieve a modest weight loss that would improve glycemic control, lipids, BP, and other cardiovascular risk factors [26]. Improvement in referral and dietary and exercise counseling are priorities for all people with diabetes in the future.

Other clinical indicators were at high levels, including lipids checked (92.8%, *p* < 0.00001) more than 58.3% of the BRFSS target, BP measurement (97.5%), and BMI documented (97.6%). Meanwhile, HBA1c checked ≥2 times/year (47.4%, *p* < 0.00001) was lower than the BRFSS target of 71.1%, and HBA1c checked <2 times/year was 50.3%. The lower percentage of patients with HBA1c checked ≥2 times/year may be attributed to many of those checked <2 times either missing their second test or starting to be seen in the NCD clinics in the last quarter of 2017.

Concerning vaccination, ADA recommends annual flu, PCV, and hep B vaccines for people with diabetes in a specific age group [1]. However, the benefits of preventive vaccination in diabetes are not very well articulated [27, 28], nor fully appreciated by health practitioners and other stakeholders, including patients. In our study, flu 40.9% (*p* < 0.00001) and PCV 43.9% (*p* < 0.00001) vaccination rates were lower than the BRFSS targets of 80% and 60%, respectively. The rate of hep B vaccination was very low, at 7.5%. These findings suggest the need for improving awareness, communication, and counseling skills among physicians and diabetes educators [29] to encourage more patients to adhere to vaccination advice.

Depression has been found to be prevalent in one in four people with type 2 diabetes. It is linked to increased risk of type 2 diabetes and its related micro- and macrovascular complications and vice versa [30]. In our study, depression screening was performed for one-quarter of patients and of those screened, 1.6% had depression. We attribute that low number to physicians' limited consultation time and minimal training. Thus, further mental health training for physicians is required to reduce the screening gap. Shortly after the study period, the PHCC implemented depression screening by nurses, to be followed up by physicians.

A higher proportion of our patients (89.6%, *p* < 0.0001) had foot examinations compared to the BRFSS target of 74.8% [11] due to the combined efforts of trained nurses who conduct initial foot examinations and physicians who subsequently review them. The rate of retinal screening was high at 72.3% (*p* < 0.0001) compared to the BRFSS target of 58.7%. Of those screened, 8.7% had retinopathy compared to the ADA's report of up to 21% of type 2 diabetes patients having retinopathy at the time of diagnosis, with most developing some degree of it over time [31]. Albuminuria screening was also higher among our patients (80.9%, *p* < 0.0001) than the BRFSS target of 37.0%. Frequent screening resulted in detection of albuminuria in 39.2% of our patients, which is comparable to the percentages having proteinuria in Saudi Arabia (54.3%), Oman (42.5%) [32], and the UK (30%) [33].

The percentage of patients with blood pressure at goal was 70.2% (*p* < 0.0001) compared to the BRFSS target of 57%. Advantages of successful BP control include prevention or delay of cardiovascular complications. Further reduction in uncontrolled BP can be achieved with more emphasis on adherence to current guidelines, promoting patient awareness and education, intensifying antihypertensive therapy, and providing prophylaxis where appropriate. Reduction in dietary sodium intake, smoking cessation, and daily moderate-intensity exercise of at least 30 minutes may help to maintain blood pressure, improve general health, and aid body weight management [1].

Most patients (73.8%, *p* < 0.0001) achieved the LDL-C control goal, compared to the BRFSS target of 58.2%, and only 4.5% of them recorded LDL-C ≥4.1 mmol/L. The ADA 2017 guidelines recommend that initiation and intensification of statin therapy be based on the risk profile rather than aiming for specific LDL-C goals and that LDL-C tests may be considered on an individual basis [1]. It is worth noting, however, that the most recent (2019) ADA guidelines do refer to the LDL-C level [34].

The percentage of patients with glycemic control meeting the HbA1c level of <7.0 was 35.5 (*p* < 0.0001), far below the BRFSS baseline value of 53.1%. However, a further 27.7% had HbA1c between 7.0 and 7.9%. Based on many recommendations, the target HbA1c of <7.0 is generally applicable while taking consideration of individual patients' conditions. For patients with other comorbid conditions and complications, an HbA1c target of <8.0 is acceptable [1]. The percentage of patients meeting the pooled targets of nonsmoking and controlled BP, LDL-C, and HbA1c was 12.4% (*p*=0.0026), comparable to the National Health and Nutrition Examination Survey (NHANES) database value of 14.0% [35].

The current study on quality of care in primary health care in Qatar is relatively new and comprehensive, covering data from almost all health centers across Qatar. The current study adds to the limited pool of studies on the same topic in the region. In conformity with Szabo's study in the UAE that found all UAE clinical care indicators exceed the US HEDIS diabetes care measure except for the HbA1c [6]; our study results also showed most clinical indicators exceed Szabo's study results [6] and the BRFSS benchmarks except for the HbA1c.

## 5. Conclusions

This study highlights the importance of documentation, the usefulness of EMR as a tool for gathering patient data in clinical practice, and the importance of primary care practice reference benchmarks for quality assurance. In the sample that we studied, blood pressure, LDL-C level, annual urine albumin creatinine ratio measurement, annual eye examination, and comprehensive foot examination were all controlled at a higher rate compared to the ADA standards, while only HbA1c was controlled at a lower rate. This suggests better adherence to current ADA standards across all health centers in Qatar.

Poor glycemic control is a cause for concern and merits a thorough review of current management strategy to identify areas for remedial action. The high rate of obesity and the relatively young age distribution of our diabetes patients are consistent with the findings in other Arab Gulf countries and raise questions about the rapid increase in diabetes risk factors at an earlier age in this population. The poor rate of referral and very low uptake of vaccination merit further review and remedial action, including patient education and empowerment, as well as more effective stakeholder engagement.

There were insufficient data to enable a more robust analysis of the impact of sociodemographic factors and gaps in lifestyle, diet and exercise prescription, counseling, and referral. We attribute these to a lack of education, training, skills, and awareness among all our diabetes care stakeholders, including patient social networks and health care professionals.

## 6. Recommendations

To address patient and healthcare professional factors contributing to gaps in quality improvement, we recommend the following:

- Better application of clinical guidelines and clinical control of comorbidities that cumulatively affect overall glycemic control, health, and wellbeing
- Regular screening and monitoring of diabetes patients and targeted personalized interventions
- Patient education, awareness, compliance, and active participation in self-management and
- A continuing education program to address gaps in education, awareness, competencies, and skills especially in diet and exercise, counseling/prescription, and depression screening to build capacity for diabetes care

## Figures and Tables

**Table 1:** Distribution of patients by their sociodemographic factors (*N* = 643).

No.	Variable	Values	Frequency (person)	Percentage
1	Sex	Male	388	60.3
		Female	255	39.7

2	Age groups	18–39 years	48	7.5
		40–64 years	461	71.7
		≥65 years	134	20.8

3	Nationality	Qatari	153	23.8
		Non-Qatari Arab	192	29.9
		Non-Qatari non-Arab	298	46.3

4	Marital status	Single	22	9.3
		Married	208	87.8
		Widowed/divorced	7	2.9
		Not documented	(*406*)	—

5	Educational level	Primary	18	50.0
		Secondary	7	19.4
		University	11	30.6
		Not documented	(*607*)	—

6	Employment status	Employed	260	63.9
		Not employed	147	36.1
		Not documented	(*236*)	—

7	Financial problems	Yes	1	5.6
		No	17	94.4
		Not documented	(*625*)	—

8	Smoking status	Yes	77	15.2
		No	430	84.8
		Not documented	(*136*)	—

**Table 2:** Distribution of patients by types of medications used and distribution of patients by clinical process indicator.

Variable	Values	Frequency	Percentage	BRFSS target	*Z*-score	*p* value
*Distribution of patients by clinical process indicator (N* *=* *643)*						
Referral to dietitians	Yes	256	39.9			
	No	386	60.1			
Diet counseling	Yes	401	37.6			
	No	242	62.4			
Exercise counseling	Yes	400	62.2			
	No	243	37.8			
Foot examined	Yes	576	89.6	74.8	5.05	<0.0001
	No	67	10.4			
Eye examination referral	Seen	465	72.3	58.7	5.93	<0.0001
	Ordered not seen	96	14.9			
	Not ordered	82	12.8			
HbA1c checked	<2/year	323	50.3	71.1	−7.47	<0.0001
	=>2/year	305	47.4			
	Never	15	2.3			
Lipid checked	Yes	595	92.8	58.3	15.14	<0.0001
	No	46	7.2			
UCR checked	Yes	520	80.9	37.0	30.50	<0.0001
	No	123	19.1			
Influenza vaccine	Given	263	40.9	80.0	−12.47	<0.0001
	Ordered-not given	66	10.3			
	Not ordered	314	48.8			
Pneumococcal vaccine	Given	282	43.9	60.0	−6.86	<0.0001
	Ordered-not given	27	4.2			
	Not ordered	334	51.9			
Hepatitis B vaccine	Given	48	7.5			
	Ordered-not given	31	4.8			
	Not ordered	564	87.7			
BP measurement	Yes	627	97.5			
	No	16	2.5			
BMI documented	Yes	621	96.6			
	No	22	3.4			
Depression	Depressed	10	1.6			
	Not depressed	143	22.2			
	Not documented	490	76.2			

*Distribution of patients by types of medications used (N* *=* *643)*						
Number of medications	0	9	1.4			
	1	145	22.5			
	2	157	24.4			
	3	228	35.5			
	4	92	14.6			
	5	10	1.6			
Metformin	Yes	578	89.9			
	No	65	10.1			
DDP-4 inhibitors	Yes	393	61.1			
	No	250	38.9			
Sulfonylurea	Yes	317	49.3			
	No	326	50.7			
Insulin	Yes	179	27.8			
	No	464	72.2			
Thiazolidinedione	Yes	65	10.1			
	No	578	89.9			
GLP-1 agonists	Yes	15	2.3			
	No	628	97.7			
SGLT-2 inhibitors	Yes	12	1.9			
	No	630	98.1			
Meglitinides	Yes	5	0.8			
	No	638	99.2			
Aspirin	Yes	230	35.8			
	No	413	64.2			
Statin	Yes	509	79.2			
	No	134	20.8			
Vitamin D	Yes	442	68.7			
	No	201	31.3			
Antihypertensive	ACE/ARBS	435	67.7			
	Others	38	5.9			
	No	170	26.4			

DDP-4 inhibitors: inhibitors of dipeptidyl peptidase 4; GLP-1: glucagon-like peptide-1 receptor; SGLT-2: sodium-glucose cotransporter-2; ACE/ARBs: angiotensin-converting enzyme/angiotensin receptor blockers; UCR: urinary creatinine ratio; BP: blood pressure; BMI: body mass index.

**Table 3:** Distribution of patients (*N* = 643) by clinical outcome indicators against BRFSS, CDC/NCCDPHP (2008) benchmark.

No.	Variable	Values	Frequency	PHCC results (%)	BRFSS baseline 2008 (%)	BRFSS target by 2020 (%)	*z*-score	*p* value
1.	HbA1c (%)	<7.0%	223	35.5	53.1	NA	−8.49	<0.0001
		7.0–7.9%	174	27.7	—	—		
		8.0–8.9%	100	15.9	—	—		
		9.0–9.9%	67	10.7	—	—		
		≥10.0%	64	10.2	—	—		
2.	BP control (<140/90)	Controlled	440	70.2	51.8	57.0	9.10	<0.0001
		Uncontrolled	187	29.8	—	—		
3.	LDL level (mmol/L)	<2.6	397	73.8	53.0	58.3	9.52	<0.0001
		2.6–4.0	117	21.7	—	—		
		≥4.1	24	4.5	—	—		
4	Annual U-ACR measurement ([albuminuria (UCR <3.0) (mg/mmol)]			80.9	33.6	37.0	36.80	<0.0001
		Present	204	39.2	—	—		
		Absent	316	60.8	—	—		
5.	Annual eye examination (retinopathy)	Present	56	72.3 8.7	53.4	58.7	9.06	<0.0001
		Absent	587	91.3	—	—		
6	Comprehensive foot examination			89.6	68.0	74.8	8.11	<0.0001
7.	Vitamin D level (ng/ml)	Normal (≥30)	90	17.5	—	—		
		Insufficient (20–29)	223	43.4	—	—		
		Deficient (<20)	201	39.1	—	—		
8.	Pooled target (HBA1c, LDL-C, BP)^*∗*^		—	12.4	14.0^*∗∗*^	NA	−3.01	0.0026

U-ACR: urine albumin creatinine ratio; LDL-C: low-density lipoprotein cholesterol; HbA1c: glycated hemoglobin; BP: blood pressure; BRFSS: Behavioral Risk Factor Surveillance System, CDC/NCCDPHP, 2008; PHCC: Primary Health Care Corporation. ^*∗*^Among nonsmokers. ^*∗∗*^Source: American Diabetes Association (ADA) 2017 value [15] rather than (BRFSS), CDC/NCCDPHP [11].

## Data Availability

The data that support the findings of this study are available on reasonable request from the corresponding author. The data are not publicly available as they contain information that could compromise the privacy of research participants.

## Additional Points

*Limitations*. Limited experience with a new EMR system may have contributed to inconsistencies in documentation and gaps especially in sociodemographic and lifestyle data. Inherent weaknesses of the current EMR tool may also have affected data consistency and quality. The eligibility criteria did not take into account the intervals between patient visits, nor did it distinguish patients seen at walk-in clinics for medication refills only, or the length of the visit. An extensive period of investigation would improve the representativeness and reliability of the data.
