# Corrigendum to “Indicators of Quality of Clinical Care for Type 2 Diabetes Patients in Primary Health Care Centers in Qatar: A Retrospective Analysis”

**DOI:** 10.1155/2020/2901538

**Published:** 2020-10-31

**Authors:** Saleh Attal, Mohamed H. Mahmoud, Muna Taher Aseel, Ady Candra, Paul Amuna, Mohamed Elnagmy, Mostafa Abdallah, Nahed Ismail, Ahmed Hanfy, Dia Albaw, Abdulsalam Albashir, Hisham Elmahdi

**Affiliations:** ^1^Family Medicine Residency Program, Primary Health Care Corporation, West Bay Training Center, Doha, Qatar; ^2^Research Department, Primary Health Care Corporation, Doha, Qatar

In the article titled “Indicators of Quality of Clinical Care for Type 2 Diabetes Patients in Primary Health Care Centers in Qatar: A Retrospective Analysis” [1], the name of the author Ahmed Hanfy was given incorrectly as Ahmed Abdelrazek. The revised authors' list is shown above.

## References

[1] Attal S., Mahmoud M. H., Taher Aseel M. (2019). Indicators of quality of clinical care for type 2 diabetes patients in primary health care centers in Qatar: a retrospective analysis. *International Journal of Endocrinology*.

